# Deep‐learning enabled smart insole system aiming for multifunctional foot‐healthcare applications

**DOI:** 10.1002/EXP.20230109

**Published:** 2023-11-23

**Authors:** Yu Tian, Lei Zhang, Chi Zhang, Bo Bao, Qingtong Li, Longfei Wang, Zhenqiang Song, Dachao Li

**Affiliations:** ^1^ State Key Laboratory of Precision Measuring Technology and Instruments Tianjin University Tianjin China; ^2^ CAS Center for Excellence in Nanoscience Beijing Institute of Nanoenergy and Nanosystems Chinese Academy of Sciences Beijing People's Republic of China; ^3^ School of Material Science and Engineering Georgia Institute of Technology Atlanta Georgia USA; ^4^ NHC Key Laboratory of Hormones and Development (Tianjin Medical University), Tianjin Key Laboratory of Metabolic Diseases Tianjin Medical University Metabolic Diseases Hospital and Tianjin Institute of Endocrinology Tianjin China

**Keywords:** 1D‐CNN, capacitive pressure sensor, deep‐learning, foot healthcare, smart insole system

## Abstract

Real‐time foot pressure monitoring using wearable smart systems, with comprehensive foot health monitoring and analysis, can enhance quality of life and prevent foot‐related diseases. However, traditional smart insole solutions that rely on basic data analysis methods of manual feature extraction are limited to real‐time plantar pressure mapping and gait analysis, failing to meet the diverse needs of users for comprehensive foot healthcare. To address this, we propose a deep learning‐enabled smart insole system comprising a plantar pressure sensing insole, portable circuit board, deep learning and data analysis blocks, and software interface. The capacitive sensing insole can map both static and dynamic plantar pressure with a wide range over 500 kPa and excellent sensitivity. Statistical tools are used to analyze long‐term foot pressure usage data, providing indicators for early prevention of foot diseases and key data labels for deep learning algorithms to uncover insights into the relationship between plantar pressure patterns and foot issues. Additionally, a segmentation method assisted deep learning model is implemented for exercise‐fatigue recognition as a proof of concept, achieving a high classification accuracy of 95%. The system also demonstrates various foot healthcare applications, including daily activity statistics, exercise injury avoidance, and diabetic foot ulcer prevention.

## Graphical abstract

This paper proposes a deep‐learning enabled wearable smart insole system. With a highly sensitive capacitive pressure sensing insole and deep‐learning enabled data analysis process, it provides long‐term data analysis, early prevention indicators, and deep insights into the relationship between plantar pressure and foot issues. Various foot‐healthcare applications are proven, including daily statistics, exercise injury avoidance, and diabetic foot ulcer prevention.

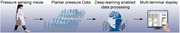

## 1 INTRODUCTION

**FIGURE 1:**
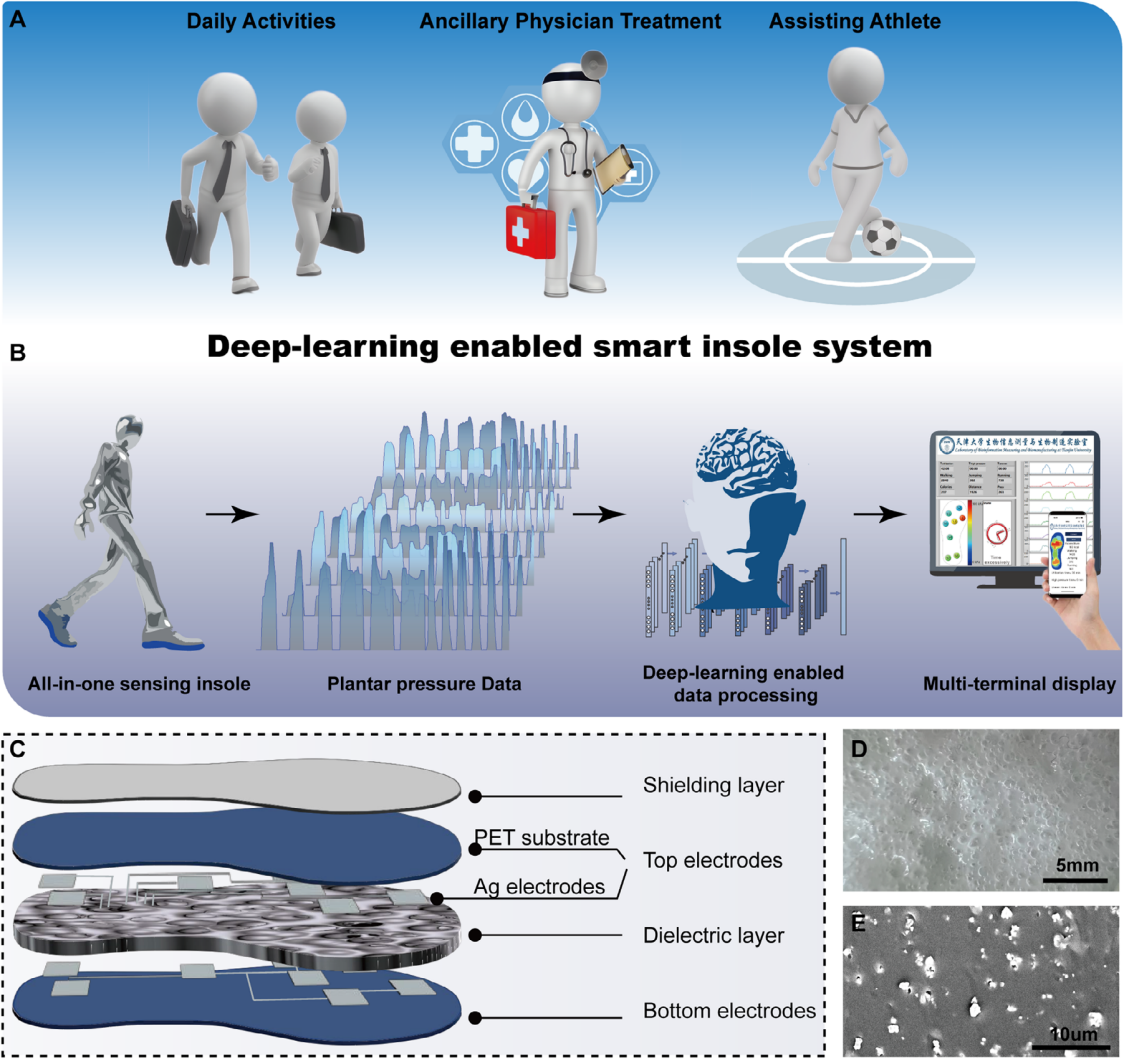
The deep‐learning enabled smart insole system configuration. (A) Schematics of smart insole system for realizing multifunctional foot‐healthcare applications for users. (B) Schematics of deep learning enabled real‐time pressure mapping smart insole system. (C) Basic structure of all‐in‐one capacitive pressure sensing matrix, which consists of four layers: shielding layer, top electrodes of the 8‐channel capacitive pressure sensors, porous BTO@PDMS dielectric layer, bottom electrodes connected to the ground. (D) Microscopic and (E) SEM cross‐sectional images of porous BTO@PDMS dielectric film.

Smart systems have garnered attention for monitoring physiological signals in humans, facilitating early disease diagnosis and prevention.^[^ ^1^ ^]^ The human foot, being a vital body part, bears the weight and pressure of the body while providing support for movement. Real‐time plantar foot pressure measurement and analysis, as depicted in Figure 1A, can rectify gait and posture issues, avert foot diseases, provide tailored rehabilitation plans for patients with foot ailments, and aid athletes in maximizing athletic performance while minimizing the risk of injuries.^[^ ^2^ ^]^ Force platforms or force plates are a conventional measuring tool widely used in hospitals and professional institutions to quantify and analyze plantar pressure.^[^ ^3^ ^]^ Despite their high measurement accuracy, the limitations of high cost, poor portability, and the requirement for professional knowledge and training to interpret the data make it challenging to use them for real‐time foot pressure data collection in everyday life, which hampers long‐term personal health management. The smart insole system is considered as an effective solution by the advantages of conformability, low cost, and intuitiveness, that integrates flexible pressure sensors, signal acquisition and processing modules, and wireless transmission modules into wearable insoles. Many efforts have been devoted to the development of smart insole systems in recent years. For instance, Tao et al.^[^ ^4^ ^]^ designed a capacitive pressure mapping smart insole system that can realize both static and dynamic real‐time plantar pressure mapping. Lin et al.^[^ ^5^ ^]^ developed a triboelectric smart insole to monitor various gait patterns. Generally, the flexible pressure sensors utilize mechanisms such as capacitance,^[^ ^6^ ^]^ resistance,^[^ ^7^ ^]^ piezoelectricity,^[^ ^6^, ^8^ ^]^ and triboelectricity.^[^ ^9^ ^]^ Among them, piezoelectric and triboelectric pressure sensors are well‐known for their high sensitivity and rapid response, but they are unable to measure static pressure. Resistive pressure sensors generally exhibit slow response speeds in dynamic pressure measurement and are susceptible to the creep phenomenon, making them unsuitable for long‐term measurements. Thus, the advantages of high sensitivity, rapid response, low power consumption, and the ability to measure both static and dynamic pressure make capacitive sensors the best candidates for a smart insole system.

Current research on wearable smart insole systems is mainly focused on developing high‐performance or highly integrated flexible pressure sensors matrix for real‐time plantar pressure mapping and gait analysis.^[^ ^4^, ^5^ ^]^ However, their data analysis methods are relied on simple manual feature extraction, such as amplitude, frequency, and peak count, leading to substantial feature loss of foot pressure information. Therefore, it is difficult to establish effective correlation models between real‐time plantar pressure or gait information and foot‐related diseases or sports injuries. This also makes it challenging to achieve multifunctional foot healthcare using smart insole systems.^[^ ^10^ ^]^ Deep learning has recently revolutionized intelligent data analysis across various disciplines.^[^ ^9^, ^11^ ^]^ With its impressive generalization capabilities, it can automatically extract intricate features and patterns from large volumes of sensor data, eliminating the need for manual design. The integration of deep learning with wearable sensors has proven to unlock a wider range of diverse and complex information, surpassing the limitations of traditional manual feature extraction methods. For example, Wen et al.^[^ ^12^ ^]^ proposed a deep learning‐enabled sign language recognition system by integrating sensing gloves and deep‐learning block and achieved an accuracy of up to 95% for sign language content recognition. Fang et al.^[^ ^13^ ^]^ designed a machine learning‐assisted smart face mask for chronic respiratory disease diagnosis and achieved an accuracy of 100% for respiration pattern classification. Therefore, combining deep learning algorithms with the wearable smart insole system can achieve more accurate and personalized foot health monitoring and exercise analysis, providing users with better health management and exercise experience.

In this study, a deep‐learning enabled smart insole system is developed for multifunctional foot‐healthcare applications. Firstly, a capacitive pressure sensing matrix based on porous  BaTiO_3_@PDMS (BTO@PDMS) dielectric film is fabricated, capable of realizing both the static and dynamic plantar pressure mapping with the wide pressure response range of 0.4–500 kPa and high sensitivity of 0.0126 kPa^−1^ in the low‐pressure region (0–200 kPa) and 0.0038 kPa^−1^ in the high‐pressure region (200–500 kPa). Subsequently, the 1D‐CNN deep learning algorithm and several data statistical tools are utilized to deeply mine and analyze plantar pressure information related to foot disease prevention and evaluate abnormality of gait (classification accuracy: 95%). Finally, several foot healthcare applications are demonstrated by our system‐level smart insole system that integrates the as‐fabricated plantar pressure sensing matrix, a portable circuit board, data analysis tools including algorithms, and a software interface.

## 2 RESULTS AND DISCUSSION

### 2.1 The deep learning enabled smart insole system configuration

Figure 1B shows the schematic diagram of the smart insole system, which consists of an integrated insole‐shaped capacitive pressure sensing matrix that converts plantar pressure into capacitance signals. These signals are then collected in real‐time by a portable signal acquisition circuit and wirelessly transmitted to various terminal devices equipped with deep learning algorithms. This system not only can detect real‐time plantar pressure and posture, statistics daily activity data, but also can deeply mine the foot utilization data to provide users with valuable insights for foot disease prevention and an improved sports experience.

The capacitive pressure sensing matrix includes eight integrated capacitive pressure sensors (CPS), is composed of a shield layer, top and bottom electrodes, and a dielectric layer (Figure 1C). Detailed preparation process is provided in the Experimental Section. Briefly, the shield layer is made of conductive fabric to directly contact the foot, which has excellent biocompatibility, breathability, and conductivity. The bottom and top electrodes are fabricated by screen‐printing conductive silver ink onto a PET substrate. The dielectric layer utilizes a porous BTO@PDMS film prepared by adding HCL to a mixture of PDMS, BaTiO_3_, and NaHCO_3_, which then generates porous structure in the BaTiO_3_@PDMS elastomer. The use of porous structures and doped high dielectric constant nanoparticles is a crucial method for enhancing the sensing performance of capacitive pressure sensors.^[^ ^14^ ^]^ Microscopic and Scanning Electron Microscope(SEM) images (Figure 1D and Figure S1) illustrate the porous structure of the BTO@PDMS dielectric layer. Moreover, Figure 1E and Figure S2 demonstrate that BaTiO_3_ nanoparticles are uniformly dispersed throughout the PDMS matrix, the black background represents the PDMS substrate, while the white particles represent the uniformly dispersed BaTiO_3_ nanoparticles.^[^ ^15^ ^]^

### 2.2 Optimization and characterization of CPS

**FIGURE 2:**
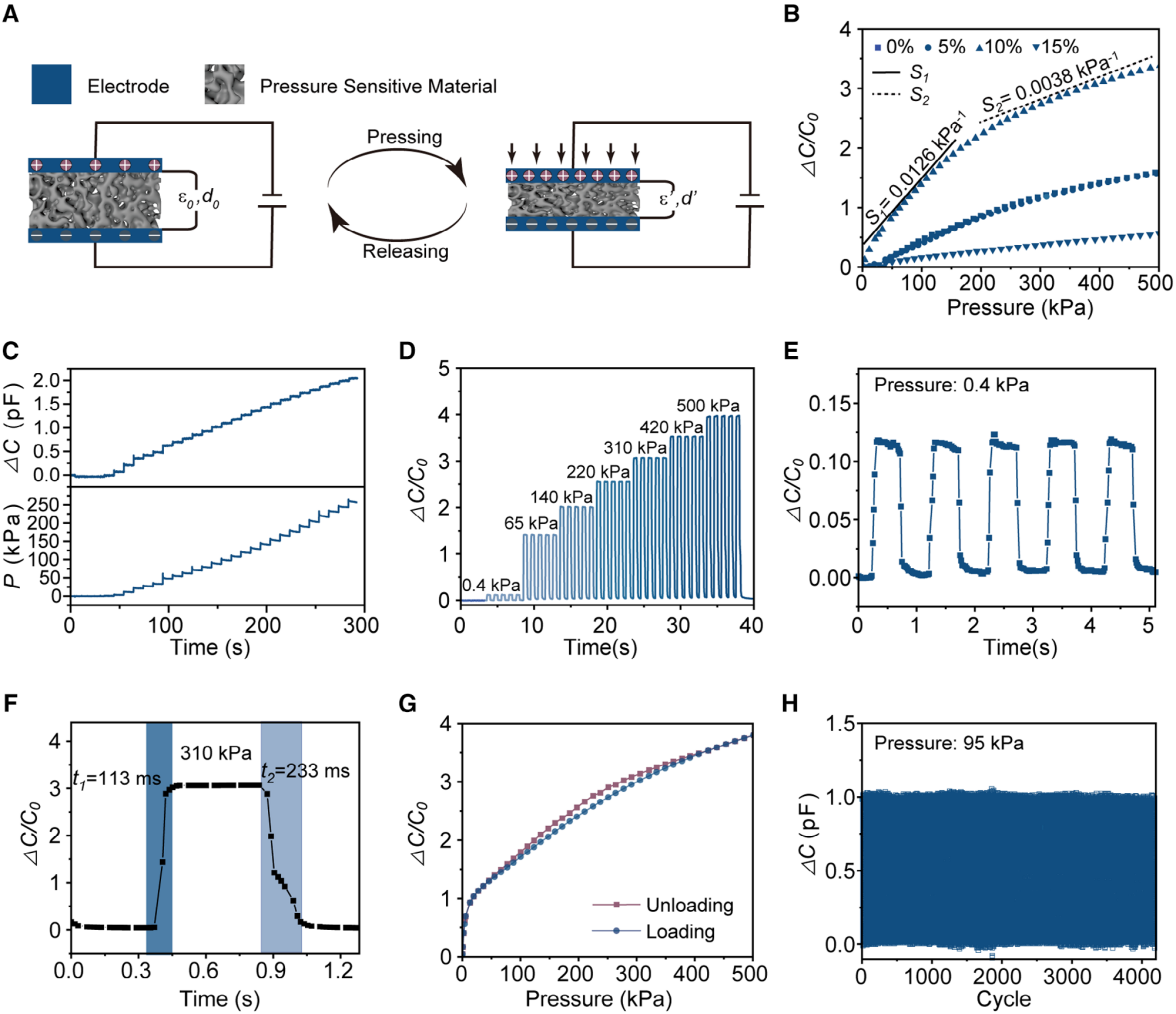
Optimization and characteristics of capacitive pressure sensor (CPS). (A) Working principle of the CPS. (B) Relative capacitance variation‐pressure curve for different additive BaTiO_3_ content (0, 5%, 10%, 15% wt%) in the range of 0∼500 kPa. (C) Real‐time capacitance variation and the corresponding applied stepped pressure‐time curves. (D) Relative capacitance variation at different external pressures (0.4, 65, 140, 220, 310, 420, and 500 kPa). (E) Relative capacitance variation at the pressure detection limit of 0.4 kPa. (F) Response and recovery time of the CPS for rising and falling loads. (G) Hysteresis response of CPS. (H) 4000‐cycle repeatability of the sensor under an applied pressure of 95 kPa.

The capacitive pressure sensor (CPS) detects external applied pressure by measuring the capacitive change. Capacitance can be defined as follows:^[^ ^16^ ^]^

(1) $$C\;=\frac{\varepsilon A}{d}\;$$

where *C* is the capacitance of the CPS, *ε* is the permittivity of dielectric layer, *A* is the contact area, and *d* is the thickness of dielectric layer, respectively. For this work, the permittivity of porous BTO@PDMS dielectric film can be calculated as follows:

(2) $$\varepsilon \;=\varepsilon_{air}\;V_{air}+\varepsilon_{BTO@PDMS}V_{BTO@PDMS}$$

where $\varepsilon_{air}$ and $\varepsilon_{BTO@PDMS}$ are the permittivity of air ($\varepsilon_{air\;}$ = 1) and BTO@PDMS, $V_{air}$ and $V_{BTO@PDMS}$ are the volume fraction of air and BTO@PDMS, respectively. As external forces are applied to the CPS, the dielectric film thickness decreases while the permittivity increases (Figure 2A). Moreover, the porous structure of BTO@PDMS film can increase the surface area of dielectric layer. These characteristics will significantly enhance the sensing performance of the CPS.

To optimize the performance of the CPS, we conduct further research by adjusting the additive content of BaTiO_3_ nanoparticles (0%, 5%, 10%, 15%) and the thickness of the dielectric layer (1.45, 2.2, 3 mm). Figure 2B shows the static relative capacitance variation of the CPS with different BaTiO_3_ additive content. Sensitivity is a crucial parameter for evaluating the performance of CPS and is defined as $S\;=\frac{C-C_{0}}{C_{0}\Delta p}\;$, where *C* and *C*_0_ are the capacitance of the CPS with and without the external applied pressure, and Δp is the variation of external applied pressure. For all the CPS devices, the sensitivity is initially constant and gradually decreases at the higher‐pressure region. This is a common phenomenon for porous dielectric materials, as the deformation becomes more limited under a higher applied pressure. The CPS with 10% BaTiO_3_ additive content output a higher capacitance under same pressure and present a maximum sensitivity. This may be attributed to the higher additive content of BaTiO_3_ nanoparticles enhancing the permittivity but reducing the elastic modulus of the composite dielectric layer.^[^ ^17^ ^]^ Figure S4 demonstrates that the CPS exhibited higher sensitivity with a thinner dielectric thickness. Considering the structural stability and the wearing comfort for users, we prefer the porous BTO@PDMS dielectric layer with 10% BaTiO_3_ additive content and 1.45 mm thickness to be the optimal choice for subsequent CPS characterization.

The optimized CPS present a wide detection range of up to 500 kPa (Figure 2B). The sensitivity is 0.0126 kPa^−1^ in the low‐pressure region (0–200 kPa) and 0.0038 kPa^−1^ in the high‐pressure region (200–500 kPa), respectively. Moreover, Figure 2C, exhibit the relative capacitance variation of CPS at different static loading pressures and dynamic applied pressure ranging from 0.4 to 500 kPa. These results highlight the superior consistency and stability of the CPS, confirming its feasibility for continuous perception of both static and dynamic pressure. Furthermore, Figure 2E–H demonstrate that the CPS has a minimum pressure detection limit of 0.4 kPa, response and recovery times of 113 and 233 ms, a degree of hysteresis of 4.8%, and excellent durability demonstrated by 4000‐cycle repeatability under an applied pressure of 95 kPa. These exceptional properties, including a wide detection range, high sensitivity, excellent stability, and fast response, make the CPS highly suitable for plantar pressure monitoring applications.

### 2.3 Real‐time plantar pressure acquisition and mapping

**FIGURE 3:**
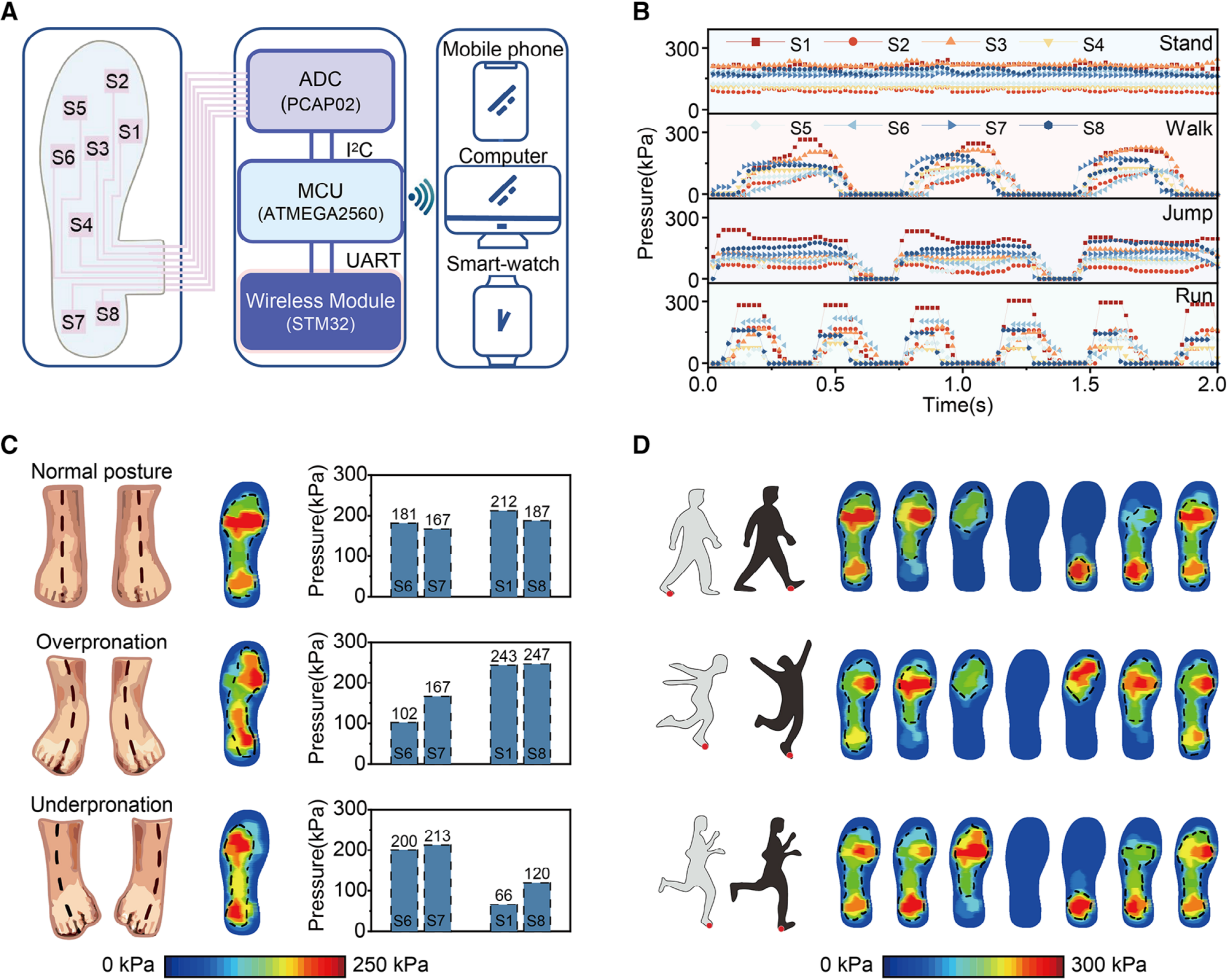
Real‐time plantar pressure acquisition and mapping based on smart insole system. (A) Circuit diagram displaying the signal flow in the smart insole system including the acquired analog capacitive signal from all‐in‐one pressure sensing matrix, the converted digital signal from DAQ circuit with the wireless transmitter, and the acquisition and mapping results shown in multiple terminals with the wireless receiver. (B) Pressure response curves of 8‐channel CPS under the postures of standing, walking, jumping, and running. (C) Thermodynamic diagram of three static standing postures (normal posture, underpronation, and overpronation), and the corresponding pressure histogram of S6, S7 (left part) and S1, S8 (right part). (D) Dynamic pressure mapping for walking, jumping, and running, respectively.

To achieve real‐time high‐precision acquisition of plantar pressure information, a simple and effective method is to achieve it by integrating a large number of flexible pressure sensors. However, as the number of sensors increases, due to the high sensitivity of CPS, crosstalk between channels is easy to occur, and the system power consumption will also increase significantly, which will affect the detection accuracy and long‐term working capability of plantar pressure information. Therefore, to map the plantar pressure change clearly with a small number of sensors, the CPS position needs to be optimized. Kanitthika et al.^[^ ^18^ ^]^ according to plantar pressure changing trends divided the plantar area into four regions: the heel region, the metatarsal region, the toe region, and the mid‐foot contour line region. Subsequently, Wei et al.^[^ ^19^ ^]^ and Sanchis‐Sanchis et al.^[^ ^20^ ^]^ proposed a widely adopted subdivision scheme with nine regions, as shown in Figure S5, namely hallux region, rest toes region, medial metatarsus region, central metatarsus region, lateral metatarsus region, medical arch region, lateral arch region, medial heel region, and lateral heel region. Considering the lower pressure in the medial arch area, an 8‐channel CPS matrix is ultimately selected for real‐time plantar pressure acquisition (Figure 3A), including the heel region (S7, S8), the mid‐foot contour line region (S4), the metatarsal region (S1, S3, S6), and the toe region (S2, S5). A digital photo is shown in Figure S6 and the weight of the smart insole is 28 g (Figure S7). The smart insole as a whole also exhibits excellent durability by 4000‐cycle repeatability (Figure S8) and mechanical adhesion (Figure S9). As the sensing matrix converts plantar pressure into the capacitive signals, a signal acquisition circuit is designed to collect and wirelessly transmit the plantar pressure data to terminals. This circuit includes an analog‐to‐digital converter (ADC) module, a micro control unit module (MCU), and a wireless module. The diagram and optical photograph of the circuit are presented in Figures S10 and S11.

Figure 3B illustrates the plantar pressure response curves for different postures, including static standing posture and dynamic postures of walking (three times), jumping (three times), and running (five times)—fundamental gaits in daily life. Table S1 provides the basic information of the user. The plantar pressure response signals exhibit relatively independent characteristics for different postures, and the same posture shows high repeatability. During the static standing posture, the multi‐channel plantar pressure response remains almost unchanged. However, it responds to varying degrees with changes in body posture during dynamic walking, running, and jumping. Regardless of the static standing or dynamic walking, running, and jumping postures, the high plantar pressure on the foot is mainly concentrated in the metatarsal region (S1, S3, S6) and the heel region (S7, S8), respectively.

To obtain a pressure distribution mapping of the entire foot region, the B‐spline function *i*th the advantage of high accuracy, good smoothness, and high computational efficiency, is utilized to fit discrete pressure sensing signal into pressure distribution mapping surfaces through local approximation and recursive calculation. Figure 3C and Figure S12 display the plantar pressure mapping results and the corresponding pressure histogram of static normal standing, underpronation, and overpronation postures. In the normal standing posture, the plantar pressure distribution is symmetrical on both sides. However, in underpronation and overpronation postures, the plantar pressure distribution is concentrated on the right and left sides of the foot, respectively. There is a significant difference between the values of the left two sensors (S6, S7) and the right two sensors (S1, S8) in the metatarsal and heel regions. The determination of the normal standing, underpronation, and overpronation postures is based on the foot posture index method, widely used in clinical practice, as shown in Figure S13. It is worth noting that long‐term improper standing postures can lead to instability of the knee and hip joints, affecting the biomechanical characteristics of the spine and increasing the risk of spinal diseases such as lumbar disc herniation. Figure 3D displays the plantar pressure mapping results of walking, jumping, and running postures in a dynamic stance. All postures start from complete contact of the foot with the ground and end with complete contact of the foot with the ground again. The regular changes in the center of gravity can be clearly observed, which are consistent with the movements of the subjects. Moreover, we utilized a standard force plate as a gold standard device to evaluate the performance of our smart insole system. The result showed good consistency with our smart insole system, demonstrating the excellent accuracy of the smart insole system (Figures S14–S16). In summary, our smart insole system demonstrates its real‐time and highly sensitive plantar pressure mapping capabilities and is capable of distinguishing various static and dynamic gaits, including but not limited to standing, walking, running, and jumping postures.

### 2.4 Statistical analysis of plantar pressure during long‐term daily use

**FIGURE 4:**
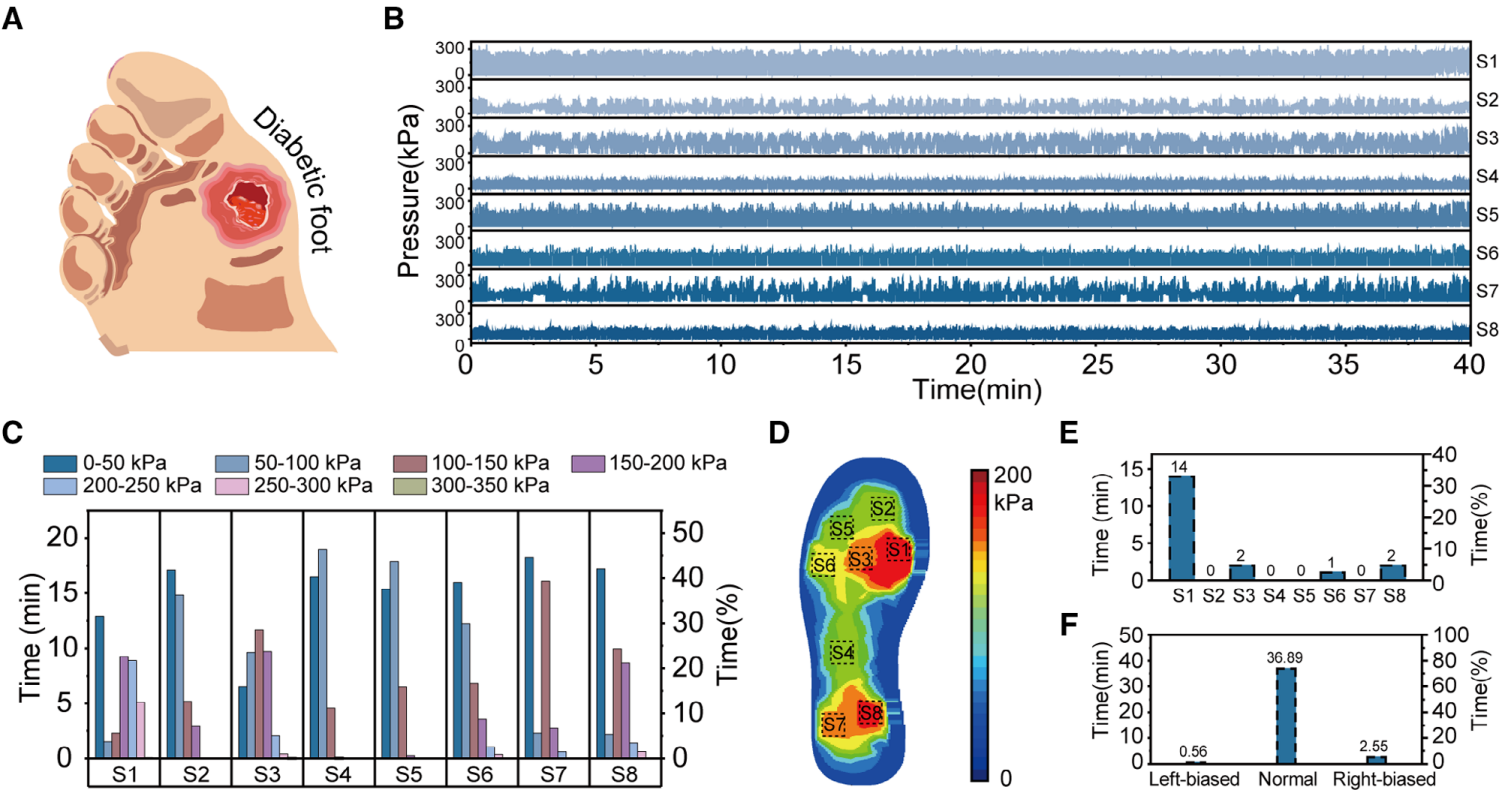
Statistical analysis of plantar pressure during daily long‐term use. (A) The statistics and analysis of long‐term plantar pressure information can detect and prevent the occurrence of the diabetic foot at an early stage. (B) Use a smart insole system to collect 40 min of prolonged plantar pressure data, including a mixture of four postures: standing, walking, running, and jumping. (C) The histogram of different regions in each pressure interval, using Find function to find the intervals by the prolonged plantar pressure data. (D) The thermodynamic diagram of pressure mapping of average plantar pressure, using Mean function to calculate the average plantar pressure in different regions by the prolonged plantar pressure data excluding the 0–50 kPa portion. (E) The histogram of the time of high plantar pressure in each area, using Find function to find the high plantar pressure interval, recorded the time and area where high plantar pressure occurred, and obtained the time of high plantar pressure in each area. (F) The histogram of the time of three static standing postures, including normal posture, underpronation, and overpronation.

In addition to the real‐time collection of plantar pressure, the statistical analysis of plantar pressure information during long‐term daily use such as the average plantar pressure and the utilization of various regions, and uneven plantar pressure distribution, is also of great significance. These can be used to prevent diabetic foot ulcers at an early stage, guide the rehabilitation training of stroke patients, as well as the treatment of scoliosis and plantar fasciitis patients.^[^ ^2a^ ^]^ Take diabetic foot ulcers as an example as shown in Figure 4A, due to long‐term hyperglycemia, the number of adult diabetes patients worldwide has reached 537 million, accounting for 10.5% of the total global population.^[^ ^21^ ^]^ Diabetic foot ulcers are one of the common complications of diabetes and featured as large patient quantity and high recurrence rate. The number of diabetic foot ulcer patients accounts for about 30% of the total number of diabetes patients, and its recurrence rate is as high as 49% within one year and is even higher at 68% within five years. Previous studies have shown that the distribution of plantar pressure in diabetic foot ulcer patients is different from that of normal people, and instantaneous pressure or excessive utilization of the foot, as well as uneven pressure distribution, can lead to the occurrence or aggravation of diabetic foot ulcers. Therefore, for diabetic patients, statistical analysis and control of daily plantar pressure usage is one of the important measures to prevent diabetic foot ulcers.

To analyze long‐term plantar pressure utilization information, we collected an over 40‐min period of irregular plantar pressure data during daily activities, including standing, walking, running, and jumping, using our smart insole system (Figure 4B). Several data analysis tools are used, such as the Find function and the Mean function. Hicks, J. H. et al.^[^ ^22^ ^]^ previously divide the plantar pressure into four levels (Table S2): namely low plantar pressure zone (0‐50 kPa), medium plantar pressure zone (50–200 kPa), high plantar pressure zone (200‐400 kPa), and very high plantar pressure zone (>400 kPa). Dividing the plantar pressure data into different intervals for segmented statistics allowed for a more comprehensive understanding of the foot load situation, visually evaluating the magnitude of the plantar load and distinguishing outlier values. Figure 4C shows the usage time in each pressure interval of different foot regions (S1–S8), revealing that the pressure of the entire foot is generally in the medium pressure zone (less than 200 kPa), consistent with actual use. Figure 4E shows the utilization rate in the high plantar pressure (>200 kPa) zone of the entire foot, with the left navicular area showing the longest duration of high plantar pressure at 14 min and 6 s, accounting for 35.15% of the entire process. The average plantar pressure mapping result of the entire foot, shown in Figure 4D, reveals that the relationship between the pressure magnitude is S1 > S8 > S3 > S7 > S6 > S5 > S4 > S2, with the participant's right navicular and right heel region experiencing larger pressure loads. To accurately reflect the effective load of the foot, this result excluded the 0–50 kPa range. Similarly, the utilization rates of three postures,^[^ ^23^ ^]^ including underpronation, normal posture, and overpronation, are displayed in Figure 4F.

These statistics results serve as not only utilization indicators of plantar pressure but also key data labels for intelligent data analysis methods, such as deep learning algorithms, to establish correlation models between plantar pressure and foot‐related diseases or sports injuries. These correlation models can provide valuable insights into the relationship between plantar pressure patterns and various foot conditions, enabling early detection and prevention of foot‐related issues.

### 2.5 Segmentation‐assisted deep learning enabling exercise‐fatigue state recognition

**FIGURE 5:**
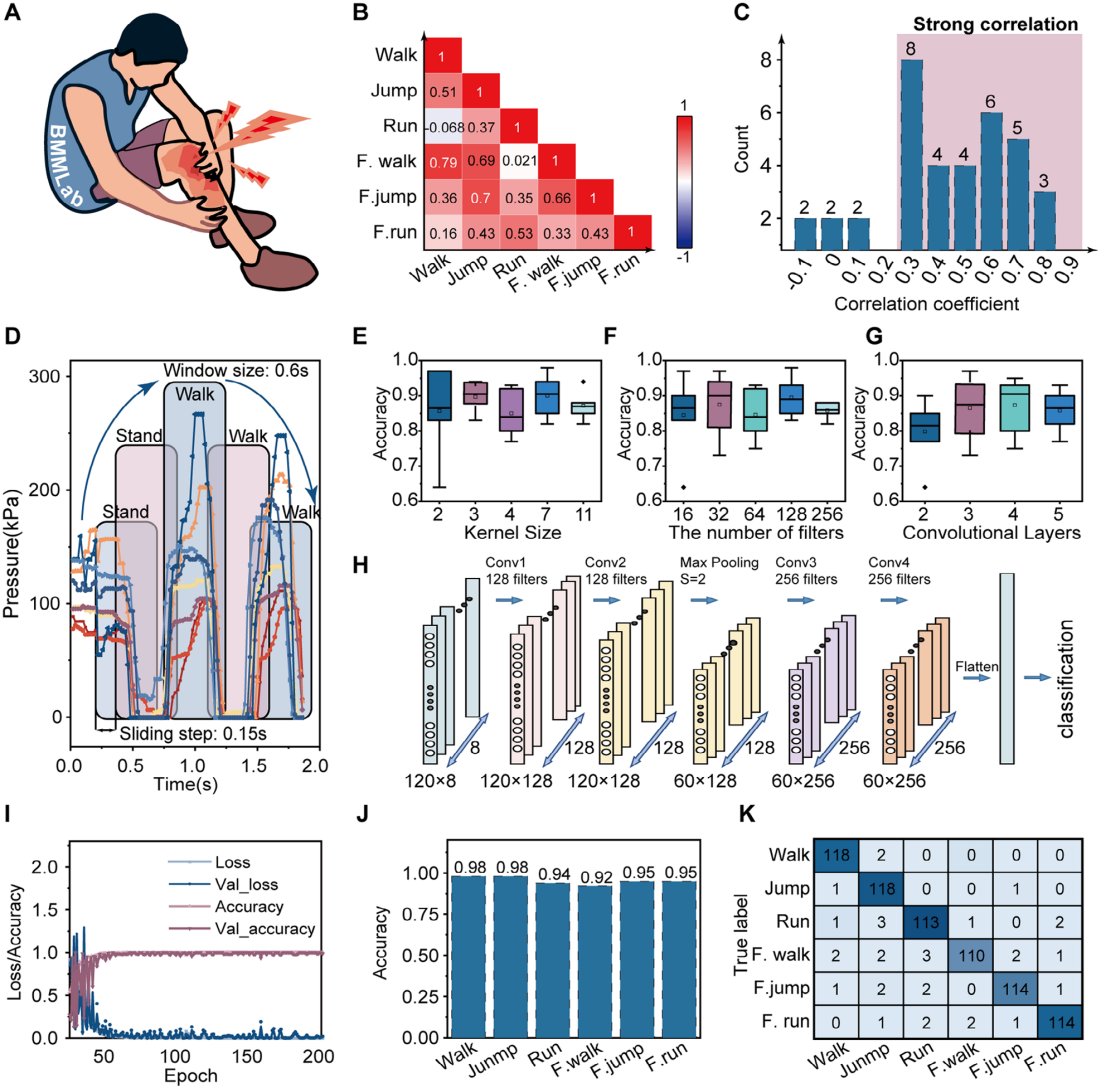
Segmentation‐assisted deep learning enabled exercise‐fatigue state recognition. (A) Real‐time detection of exercise fatigue can prevent injuries and improve training effectiveness. (B) Correlation coefficient matrix of three postures of walking, running, and jumping under normal and fatigue conditions. (C) Correlation coefficient distribution curve of three postures under normal and fatigue conditions. (D) Schematics of gait signal segmentation, ‘stand‐walk‐walk’ is taken as an example. (E–G) The structure parameters of a 1D‐CNN, including kernel size, number of filters, and number of convolutional layers, are optimized based on accuracy performance. The optimization results are visualized using boxplots that show the median, 25th and 75th percentiles (box), and 5th and 95th percentiles (whiskers), as well as outliers (single points). (H) Optimized 1D‐CNN structure. (I) Evaluation of classification accuracy, learning rate, and loss function in 200 epochs. (J) Recognition accuracy of various exercise‐fatigue states using the segmentation‐assisted 1D‐CNN algorithm. (K) Confusion matrix for three postures of walking, running, and jumping under normal and fatigue conditions.

The smart insole system enables the real‐time monitoring of plantar pressure and utilization data, which can also quantify the motion status of the human body and assess exercise fatigue levels. This is particularly beneficial for the elderly and athletes, as it can help prevent sports injuries and enhance fitness results (Figure 5A). The tentative data analysis is beneficial for the preliminary understanding of the raw data. Figure S17 displays the plantar pressure data curves between normal and fatigued states for walking, running, and jumping postures. To measure plantar pressure data under fatigued conditions, the experimenter is required to perform a specified amount of exercise. The degree of fatigue experienced by the experimenter after exercise is evaluated using the subjective feeling method as shown in Table S3, and is required generally not less than level 7. However, no significant differences are observed between signals. The most correlation coefficient falling into the strongly correlated area (>0.3) indicates high degree of similarity between postures (Figure 5B,C), which will reduce the accuracy of identifying exercise states and their fatigue levels. Therefore, a more advanced analysis method is needed to address this issue.

1D‐CNN algorithm^[^ ^11a^ ^]^ is a powerful time‐series deep learning analysis method based on convolutional neural networks that can handle multi‐channel data and automatically learn spatial and temporal features in time‐series data, while also avoiding overfitting and improving data utilization and classification accuracy. To enhance recognition accuracy of gait and fatigue states by the 1D‐CNN model, six sets of the plantar pressure data were collected including walking, jumping, and running in normal and fatigued states. Each posture consists of 150 gait cycles (120 data points per cycle), and we use a segmentation window method to process the data, dividing 18,000 data points into 600 samples. Each posture has 600 samples, of which 480 samples (80%) are used for training and 120 samples (20%) are used for testing. In addition, considering that the collected samples are all complete gait cycles of walking, running, and jumping. Whereas, in actual exercise, people may have irregular postures and incomplete gait cycles as input. As presented in Figure 5D, an irregular standing‐walking‐walking posture process occurs within a 2‐s period. A segmentation method is introduced to divide the signals of all postures into segments using a sliding window, with a sliding window of 0.6 s (120 data points) and a sliding step of 0.15 s (30 data points). When the sliding window contains a complete posture signal, the signal is labelled with the corresponding posture number. When the sliding window contains mixed posture signals (including two postures), the label will be the number of the posture that occupies more than 50% of the sliding window size. The segmentation method improves the utilization of data and the accuracy of classification.

We further optimized the kernel size, filter number, and convolutional layers of the 1D CNN model. As shown in Figure 5E–G, the 1D CNN model with 7 kernels, 128 filters, and 4 convolutional layers exhibits the best recognition accuracy. The final schematic diagram of the 1D‐CNN model is shown in Figure 5H, the network structure consists of two convolutional layers with 128 filters, two convolutional layers with 256 filters, and a pooling layer with a step size of 2.The efficiency of the 1D‐CNN algorithm is demonstrated by the learning accuracy, loss function, and learning rate, as shown in Figure 5I. The results show that the model can achieve high classification accuracy and robustness after 200 training cycles. The average prediction accuracy is as high as 95.3% (Figure 5J). The confusion matrix of the predicted labels and the true labels in the test set is also validated, indicating that the system can recognize exercise‐fatigue status in different postures (Figure 5K).

### 2.6 Demonstration in multifunctional foot‐healthcare applications

**FIGURE 6:**
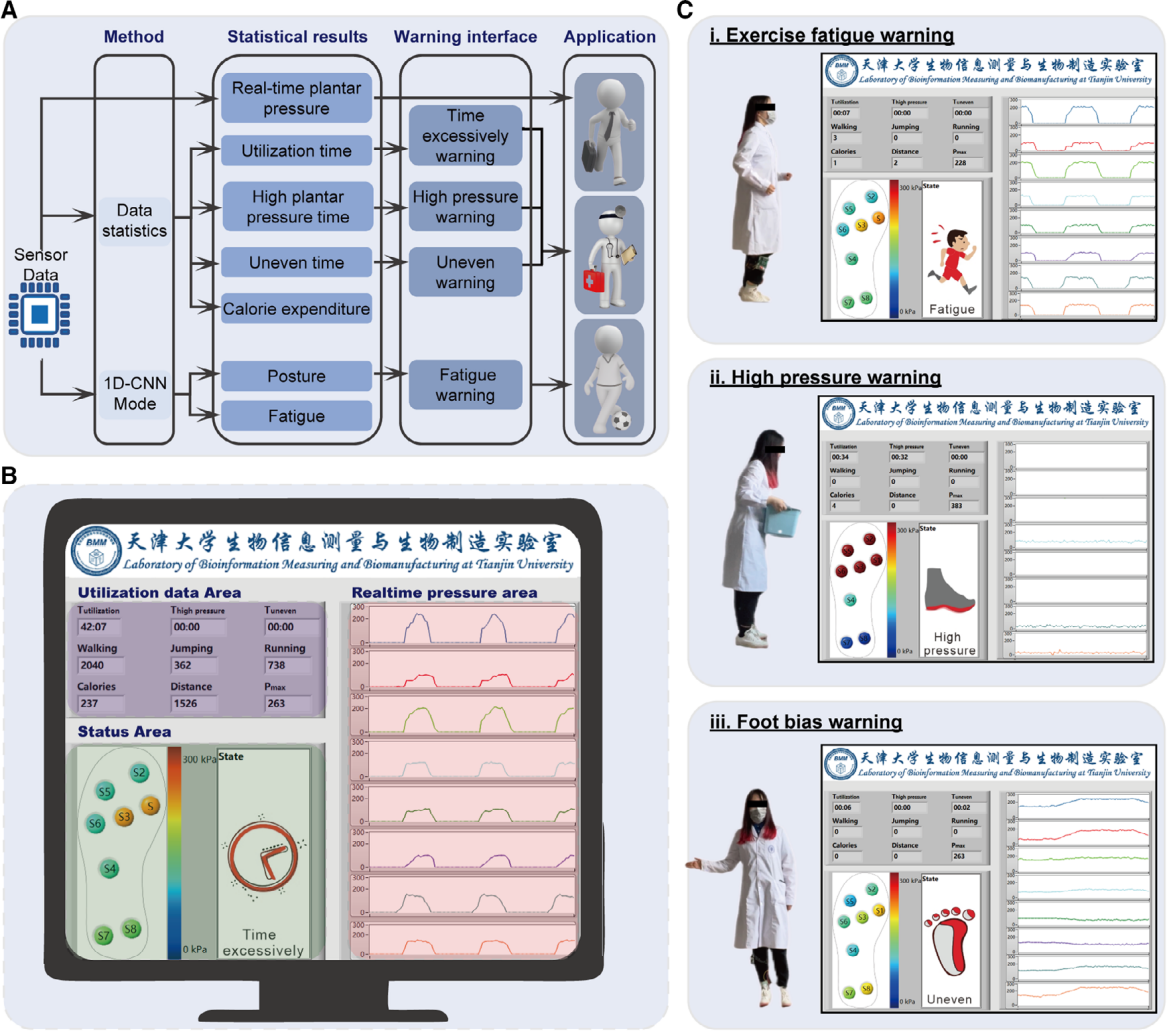
Demonstration of smart insole system for multifunctional foot‐healthcare applications. (A) Principle workflow of the deep learning enabled smart insole system. (B) The software interface on computer side includes three functional regions, namely real‐time plantar pressure curve area, statistical foot utilization information area, and status display area. (C) The issued system alert when the plantar pressure is abnormal for daily use and foot‐related diseases preventation: (i) Excercise‐fatigue warning; (ii) high plantar pressure warning; (iii) uneven foot pressure warning.

A system‐level smart insole system was developed for multifunctional foot‐healthcare applications, as shown in Figure 6A. The as‐fabricated plantar pressure sensing matrix and portable signal acquisition circuit can convert plantar pressure into capacitive signals, which are then analyzed using a deep learning algorithm and several data analysis tools. Finally, the real‐time and statistical plantar pressure data are wirelessly transmitted and displayed on a software interface of the computer terminal, as shown in Figure 6B. The software interface on the computer side, as shown in Figure 6B, is divided into three functional regions. These include (1) a real‐time plantar pressure curve area that displays the pressure curve of eight regions of the plantar in real‐time, (2) a statistical foot utilization information area that uses deep learning algorithm and statistical tools to analyze the plantar pressure information, such as the number of steps taken, calories burned, and distance travelled, and (3) a status display area that shows the real‐time pressure value of each region of the foot and the user's real‐time movement status. Furthermore, when the plantar pressure is abnormal, an alert will be issued by the system and also displayed in this area.

For everyday use, the smart insole system is primarily utilized to collect and display utilization information regarding the user's daily activities, as demonstrated in Movie S1. Figure 6B presents the statistical results of usage information after an irregular activity lasting ≈42 min, indicating that the user took 2040 steps, performed 362 jumps, ran for 738 steps, covered a distance of 1226 m, and burned 237 kcal of calories. Furthermore, to prevent exercise‐related injuries caused by fatigue, our smart insole can actively detect and alert users to their fatigue status, as depicted in Figure 6C‐i and Movie S2.

To prevent foot‐related diseases, we have given special consideration to patients with diabetic feet. Instantaneous high pressure or excessive utilization of the foot, as well as uneven pressure distribution, can increase the risk of developing or worsening diabetic foot ulcers. While ordinary individuals typically do not experience sustained high plantar pressure in their daily activities, certain specific activities such as carrying heavy objects, prolonged standing or walking, or repetitive trampling can lead to such pressure. In light of these concerns, our smart insole system offers the following features: (1) “High pressure warning”: when the user experiences an ultrahigh plantar pressure exceeding 500 kPa or a pressure lasting more than 30 s above 200 kPa, the system displays a warning and alerts the user to take necessary measures to reduce the plantar pressure, as shown in Figure 6C‐ii and Movie S3. (2) “Uneven foot pressure warning”: if the pressure difference between the left and right areas of the foot exceeds 45%, the system displays a warning and reminds the user to correct their walking posture, as shown in Figure 6C‐iii and Movie S4. (3) “Excessive use warning”: when the continuous utilization time of the foot exceeds 30 min, the system displays a warning to remind the user to take a rest, thus preventing excessive foot usage.

It is important to highlight that the deep learning‐enabled smart insole system offers a reliable and compelling platform for multifunctional foot healthcare applications. On one hand, the wearable and portable design of the plantar pressure sensing matrix and DAQ circuit board allows for the continuous collection of real‐time and statistical foot pressure data, catering to diverse application requirements. On the other hand, while the validation of the exercise‐fatigue recognition application in this study serves as a proof of concept, deep learning algorithms, as powerful intelligent data analysis tools, hold the potential to unveil the relationship between foot pressure and specific foot pathologies. Nonetheless, achieving this would necessitate the collection of extensive foot disease‐related data and the collaborative engagement of interdisciplinary research involving doctors and patients. To summarize, this study presents a potential prototype for future wearable foot healthcare systems.

## 3 CONCLUSIONS

In summary, we developed a deep‐learning enabled smart insole system aiming for multifunctional foot‐healthcare applications. Our smart insole system is capable of realizing both the static and dynamic plantar pressure mapping with the wide pressure response range of 0.4–500 kPa and high sensitivity of 0.0126 kPa^−1^ in the low‐pressure region (0–200 kPa) and 0.0038 kPa^−1^ in the high‐pressure region (200–500 kPa). Various statistical tools mainly including Find function and Mean function are used to analyze long‐term foot pressure usage data such as utilization time, high plantar pressure time, and mean foot pressure for foot disease early prevention. Moreover, the 1D‐CNN deep learning model assisted by the segmentation method is used to deeply mine and analyze plantar pressure information to determine abnormal states through foot pressure information. The validation of the exercise‐fatigue recognition as a proof of concept in this study achieves 95% classification accuracy. Finally, several foot healthcare applications are demonstrated by our system‐level smart insole system, including daily activity statistics, exercise injury avoidance, and prevention of foot ulcer in diabetes.

## 4 EXPERIMENTAL SECTION

### 4.1 Chemicals and materials

PDMS was purchased from Chemart Chemical Technology Co., Ltd. Ag ink was purchased as conductive electrode from Qingdao Nano Print Materials Technology Co., Ltd. The Eco‐Flex (00‐10) silicone was purchased from Smooth‐On Inc. The Polyethylene terephthalate film with a thickness of 0.1 mm was purchased from Tianjin Ruimekang Technology Co., Ltd. NaHCO_3_ powders (CAS: 144‐55‐8) and BaTiO_3_ nanoparticles (CAS: 12047‐27‐7) were bought from Heowns Biochem LLC, and HCl solution (38%, CAS: 7647‐01‐0) were bought from Rionlon Industry Co. Ltd. All the chemicals were directly used as received without further purification.

### 4.2 Preparation of the porous BTO@PDMS dielectric film

(1) Pour the PDMS (composed of a 10:1 ratio of PDMS elastomer and base), NaHCO_3_ powder, and BaTiO_3_ nanoparticles into a beaker with a weight ratio of 10:2:1. (2) Mechanically stir the mixture with a speed of 200 rpm for 10 min. (3) Add 6% (w/w) HCl solution into the mixture and stir for 10 min with a speed of 200 rpm. (4) Pour the mixture into a glass mould and left it for 20 min, porous BTO@PDMS film was achieved by heating in an oven at 85°C for 30 min, and different thicknesses can be controlled by the poured weight. (5) Peel off the as‐fabricated porous BTO@PDMS film from the mould. (6) Immerse the BTO@PDMS film in isopropyl alcohol and deionized water, respectively, and ultrasonic cleaning for 20 min to remove the residual NaCl nanocrystals.

### 4.3 Preparation of CPS and all‐in‐one sensing matrix

#### 4.3.1 Preparation of CPS

(1) Screen‐print Ag ink onto a flexible PET substrate as the conductive electrode with a size of 3 × 3 cm^2^. (2) Dry it on a heating platform at 120°C for 5 min. (3) Tailor the above as‐fabricated porous BTO@PDMS film with the same size of 3 × 3 cm^2^. (4) Eco‐Flex adhesive glue the top and bottom conductive Ag electrode and porous BTO@PDMS film together with a sandwich structure.

#### 4.3.2 Preparation of all‐in‐one sensing insole

The all‐in‐one sensing insole presents a similar structure with the CPS, including a bottom conductive electrode, a dielectric BTO@PDMS film, a top conductive electrode, and a shielding layer (from bottom to top). (1) Screen‐print conductive Ag ink onto a flexible PET substrate with a predesigned pattern (8 CPS) as shown in Figure 3A to achieve the arrayed top and bottom conductive electrodes with a size of 1.8 × 1.8 cm^2^. (2) Lasercut the Ag electrode@PET film with the insole shape. (3) Prepare the dielectric BTO@PDMS film by casting in 3D‐printed insole‐shaped mould. (4) Laser cut the conductive fabric (100% silver ion fibre fabrics) with the insole shape as the shielding layer, and the thickness of the shielding layer is 0.2 mm. (5) Assemble the insole‐shaped the bottom Ag electrode@PET film, dielectric BTO@PDMS film, the top Ag electrode@PET film, and the shielding layer together using the Eco‐Flex adhesive.

### 4.4 Characterization and electrical measurements

#### 4.4.1 Data acquisition of single CPS

The linear motor (Linmot E1100) applied the pressure to the CPS by controlling position, the pressure value is measured and recorded in real‐time using a Vernier dual‐range force sensor. Schematic diagram for applying force as shown in Figure S3. A precision LCR meter (Agilent E4980A) is utilized to measure the capacitive output of CPS, and the experimental data is collected by a self‐programmed software written by LabVIEW.

#### 4.4.2 Data acquisition of all‐in‐one sensing matrix

A portable circuit board was built for the data acquisition of all‐in‐one sensing insole, which mainly contains an analog‐to‐digital converter (PCAP02‐AE) module, an MCU chip (ATMEGA2560‐16AU), and a wireless module (STM32F030F4P6TR).

### 4.5 Pressure mapping simulation

Considering reducing system power consumption and avoiding signal crosstalk between channels, the all‐in‐one sensing insole owns 8 discrete CPS sensors. To analyze the pressure distribution characteristics of the whole foot, a cubic B‐spline surface interpolation method is utilized to interpolate the measured discrete pressure values to the whole foot and achieve continuity of pressure distribution. The B‐spline function can smooth the plantar pressure mapping result and show many advantages, such as convenient calculation, high precision, and high numerical stability.

### 4.6 Daily plantar pressure information statistics

MATLAB has used to statistic the collected daily plantar pressure signals. Using Find function to find the intervals by the prolonged plantar pressure data. Using Mean function to calculate the average plantar pressure in different regions. Using Find function to find the high plantar pressure interval.

### 4.7 Neural network dataset configuration

Data from 150 gait cycles (120 data points in total) for each of the six postures of walking, running, jumping, and fatigue were collected for the construction of a 4‐layer 1D‐CNN based on the segmentation method assisted with data feature extraction and automatic identification of the posture and fatigue state of the input plantar pressure signal. Before the plantar pressure measurement in the non‐fatigue state, the experimenter performs appropriate warm‐up exercises. Before performing the plantar pressure measurement in the fatigue state, the experimenter needs to perform a certain amount of exercise (running 400 m and climbing 5 flights of stairs 5 times). A segmentation window of length 120 data points in MATLAB workspace was used, sliding in steps of 30 data points, to extract signal segments. Thus, 18,000 data points are segmented into 600 samples. There are 600 samples for each pose, of which 480 samples (80%) are used for training and 120 samples (20%) are used for testing. To fine‐tune the structural parameters of the 1D‐CNN algorithm, we employed the S‐fold cross‐validation method and randomly divided the training set (80%) into five parts. The structure and flow of the training data set are illustrated in Figure S18. Such a dataset will help this paper to perform accurate classification and identification of plantar pressure signals.

## AUTHOR CONTRIBUTIONS

**Yu Tian**: Conceptualization; investigation; data curation; methodology visualization; software development; writing — original draft. **Lei Zhang**: Conceptualization; methodology; formal analysis; writing — review & editing. **Chi Zhang**: Analysis and interpretation of data. **Bo Bao**: Software development. **Qingtong Li**: Acquisition of data. **Longfei Wang**: Give professional advice. **Zhenqiang Song**: Give professional advice. **Dachao Li**: Supervision.

## CONFLICT OF INTEREST STATEMENT

The authors declare no conflicts of interest.

## Supporting information

SUPPORTING INFORMATION

SUPPORTING INFORMATION

SUPPORTING INFORMATION

SUPPORTING INFORMATION

SUPPORTING INFORMATION

## Data Availability

Research data are not shared.
