# Supplementary material for: Deep‐learning enabled smart insole system aiming for multifunctional foot‐healthcare applications
Source: Exploration (Beijing). 2023 Nov 23;4(1):20230109. doi: 10.1002/EXP.20230109 (PMC10867401; doi:10.1002/EXP.20230109)
Supplement: Supplementary file 1 — SUPPORTING INFORMATION [file EXP2-4-20230109-s005.pdf]

Supporting Information

Deep-learning enabled Smart Insole System Aiming for Multifunctional Foot-healthcare Applications

Yu Tian[+], *Lei Zhang* [+, *], *Chi Zhang, Bo Bao, Qingtong Li, Longfei Wang, Zhenqiang Song*[*], *Dachao Li*[*]

[+]Y. Tian and L. Zhang contributed equally to this work.

Y. Tian, Prof. L. Zhang, C. Zhang, B. Bao, Q. Li, Prof. D. Li

State Key Laboratory of Precision Measuring Technology and Instruments, Tianjin University, Tianjin, 300072, China.

Prof. L. Wang

CAS Center for Excellence in Nanoscience, Beijing Institute of Nanoenergy and Nanosystems, Chinese Academy of Sciences, Beijing 101400, P. R. China.

School of Material Science and Engineering, Georgia Institute of Technology, Atlanta, GA 30332, United States.

Prof. Z. Song

NHC Key Laboratory of Hormones and Development (Tianjin Medical University), Tianjin Key Laboratory of Metabolic Diseases. Tianjin Medical University Metabolic Diseases Hospital & Tianjin Institute of Endocrinology, Tianjin, China.

E-mail: zhangleitd@tju.edu.cn; dchli@tju.edu.cn; zsong@tmu.cdu.cn

Content of the Supplementary information

Figure S1. The SEM image of porous BTO@PDMS dielectric layers.

Figure S2. The EDS mapping image of Ba and Ti elements of BTO@PDMS dielectric film.

Figure S3. The schematic diagram of the linear motor for applying force.

Figure S4. Capacitance variation-pressure curves for the dielectric layer with different thickness, including 1.45, 2.2, and 3.0 mm, and the nonstructured configuration in the range of 0-500 kPa.

Figure S5. Wei *et al.*[1] and Sanchis *et al.*[2] divided the plantar area into 9 regions.

Figure S6. Optical photograph of the all-in-one insole.

Figure S7. Optical photograph of the smart insole weighing.

Figure S8. 4000-cycle repeatability of the smart insole as a whole (8 sensors)under an applied pressure of 95 kPa.

Figure S9. Cyclic friction experiment A) Using a foot-shaped plate and applied a vertical pressure of 50kg to the smart insole, simulating the weight of a person. B) Using a linear motor to move the foot-shaped plate back and forth, simulating the front and back friction of human feet on the smart insole. C) The smart insole condition comparison before and after friction.

Figure S10. Detailed diagram of the circuit board.

Figure S11. Optical photograph of the wearing demonstration.

Figure S12. The corresponding pressure histogram of three standing postures.

A) normal posture B) severe(i) and mild(ii) overpronation C) severe(i) and mild(ii) underpronation.

Figure S13. Evaluation and prediction of individual foot posture and foot and ankle function using the foot posture index[3](FPI) .The FPI is a criterion for evaluating and predicting individual foot posture and foot and ankle function in a clinical setting, including observation of the upper and lower curvature of the outer ankle (A) the position of the frontal plane of the heel bone (B) the elevation of the talonavicular region (C) the height and angle of the medial longitudinal arch (D) the visibility of the toes (E) distinguishing between five postures, including severe underpronatio(i) mild underpronatio(ii) normal posture(iii) severe overpronation(iv) and mild overpronation(v).

Figure S14. Optical photograph of the force plate (Sensor Medica).

Figure S15. Comparison results of the smart insole and the force plate, including three static standing postures (A) and walking (B).

Figure S16. Comparison of pressure values at 8 points between the smart insole and the standard force plate.

Figure S17. Capacitance response curves of the sensors as walking(A), jumping(B), and running(C) under the conditions of normal and fatigue by the experimenter.

Figure S18. The training process of the 1D-CNN algorithm.

Table S1. Hicks, J. H. *et al.*[4] classified plantar pressure into 4 levels.

Table S2. Basic information of the experimental participant.

Table S3. Rate of Perceived Exertion (RPE) Scale.

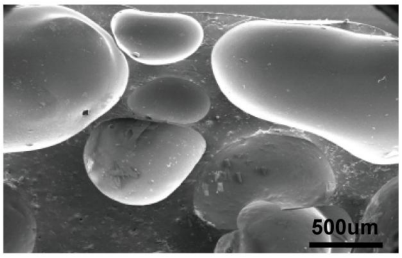

Figure S1. The SEM image of porous BTO@PDMS dielectric layers.

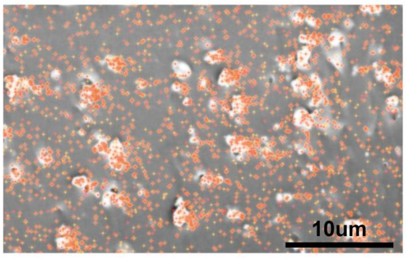

Figure S2. The EDS mapping image of Ba and Ti elements of BTO@PDMS dielectric film.

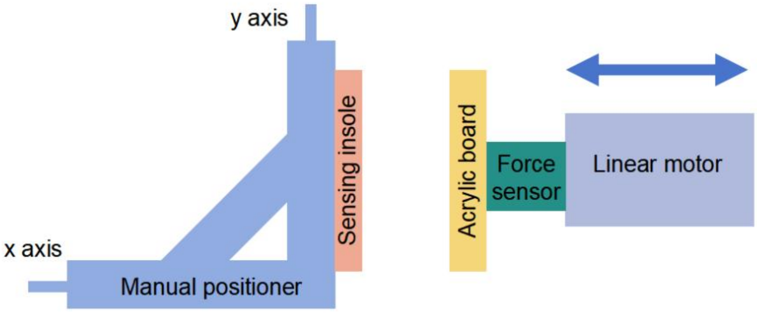

Figure S3. The schematic diagram of the linear motor for applying force.

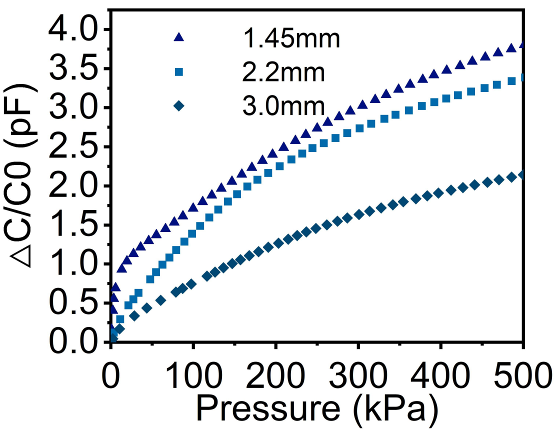

Figure S4. Capacitance variation-pressure curves for the dielectric layer with different thickness, including 1.45, 2.2, and 3.0 mm, and the nonstructured configuration in the range of 0-500 kPa.

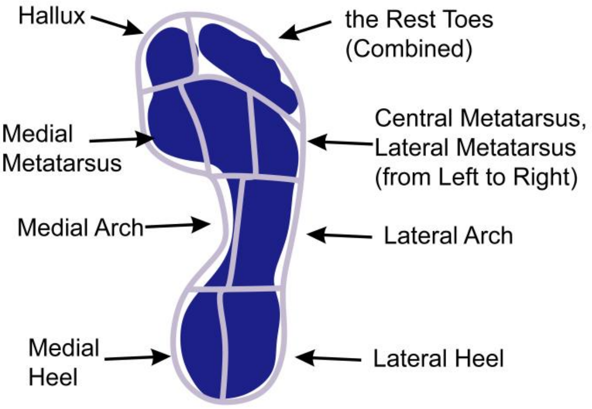

Figure S5. Wei *et al.*[1] and Sanchis *et al.*[2] divided the plantar area into 9 regions.

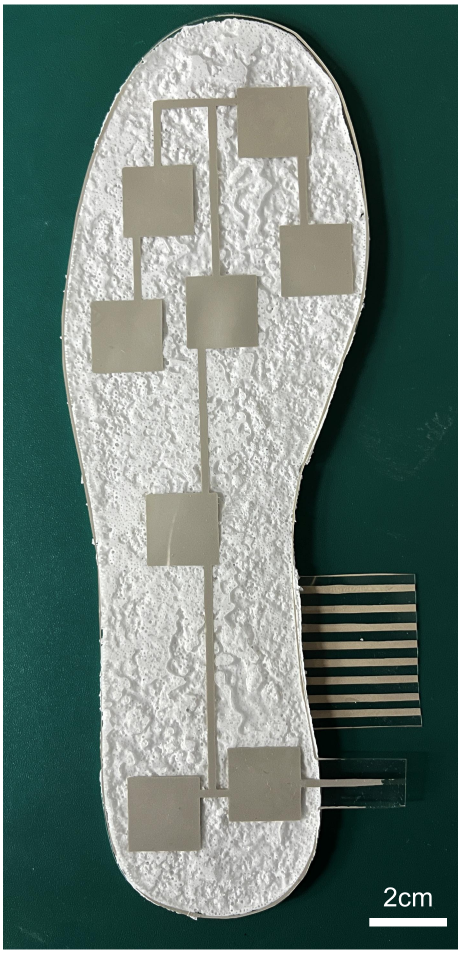

Figure S6. Optical photograph of the all-in-one insole.

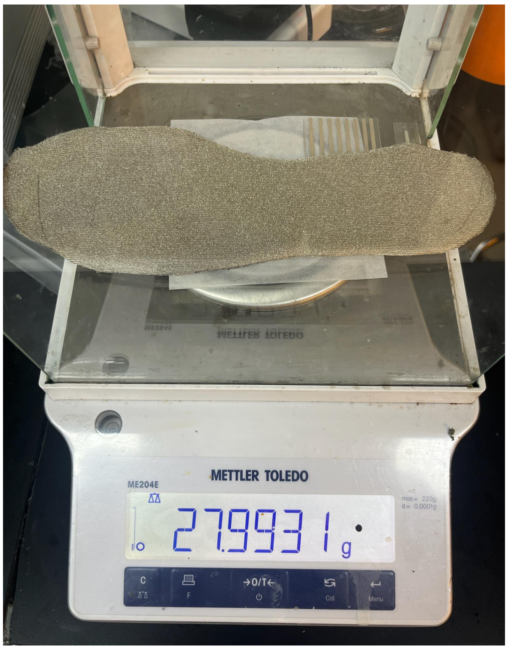

Figure S7. Optical photograph of the smart insole weighing.

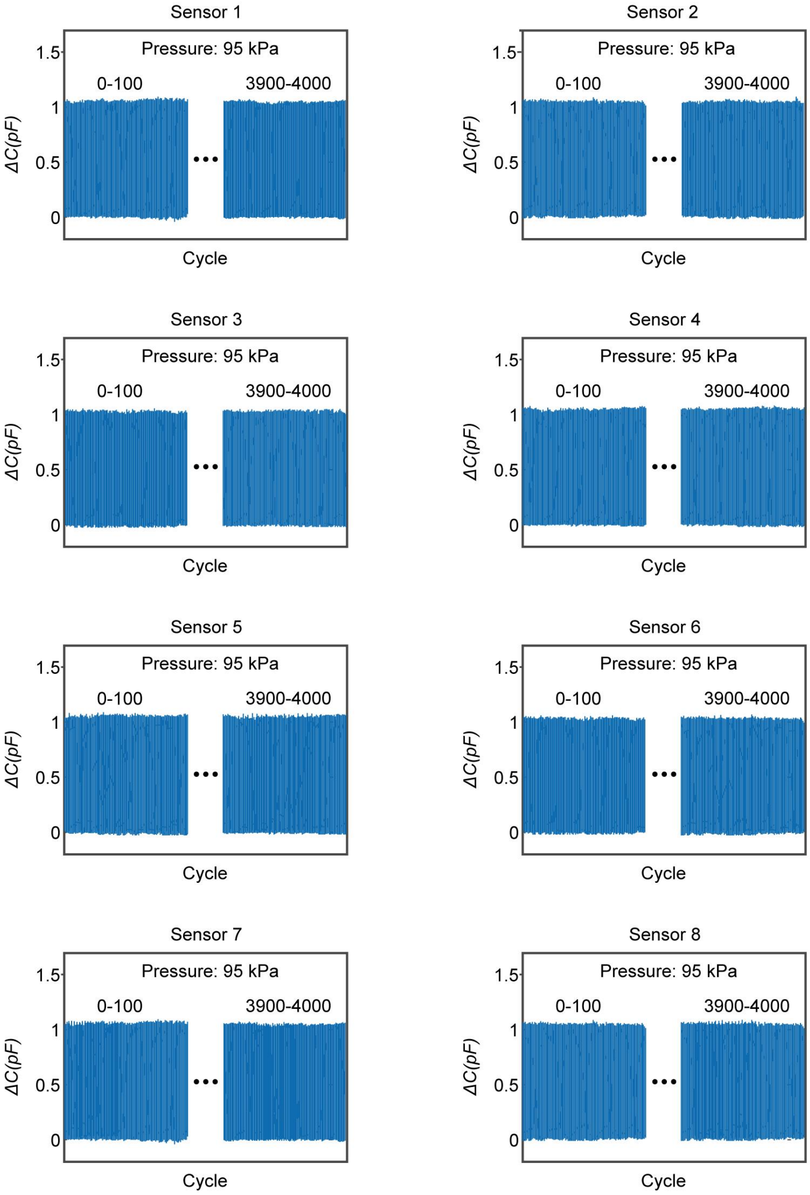

Figure S8. 4000-cycle repeatability of the smart insole as a whole (8 sensors)under an applied pressure of 95 kPa.

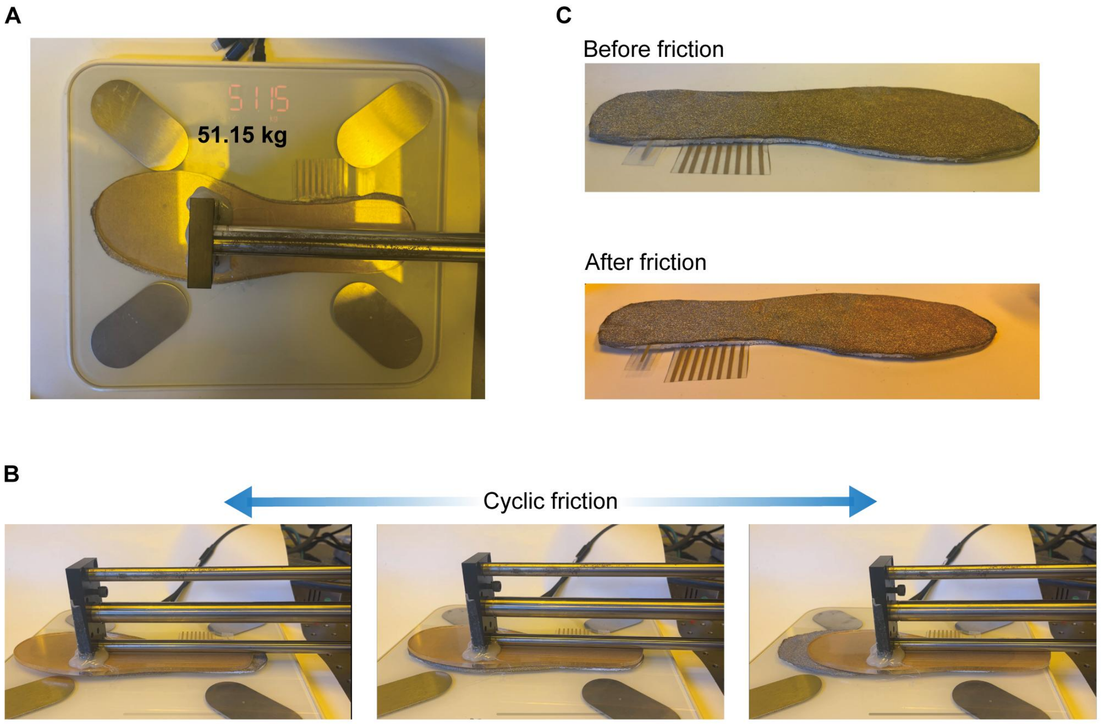

Figure S9. Cyclic friction experiment A) Using a foot-shaped plate and applied a vertical pressure of 50kg to the smart insole, simulating the weight of a person. B) Using a linear motor to move the foot-shaped plate back and forth, simulating the front and back friction of human feet on the smart insole. C) The smart insole condition comparison before and after friction.

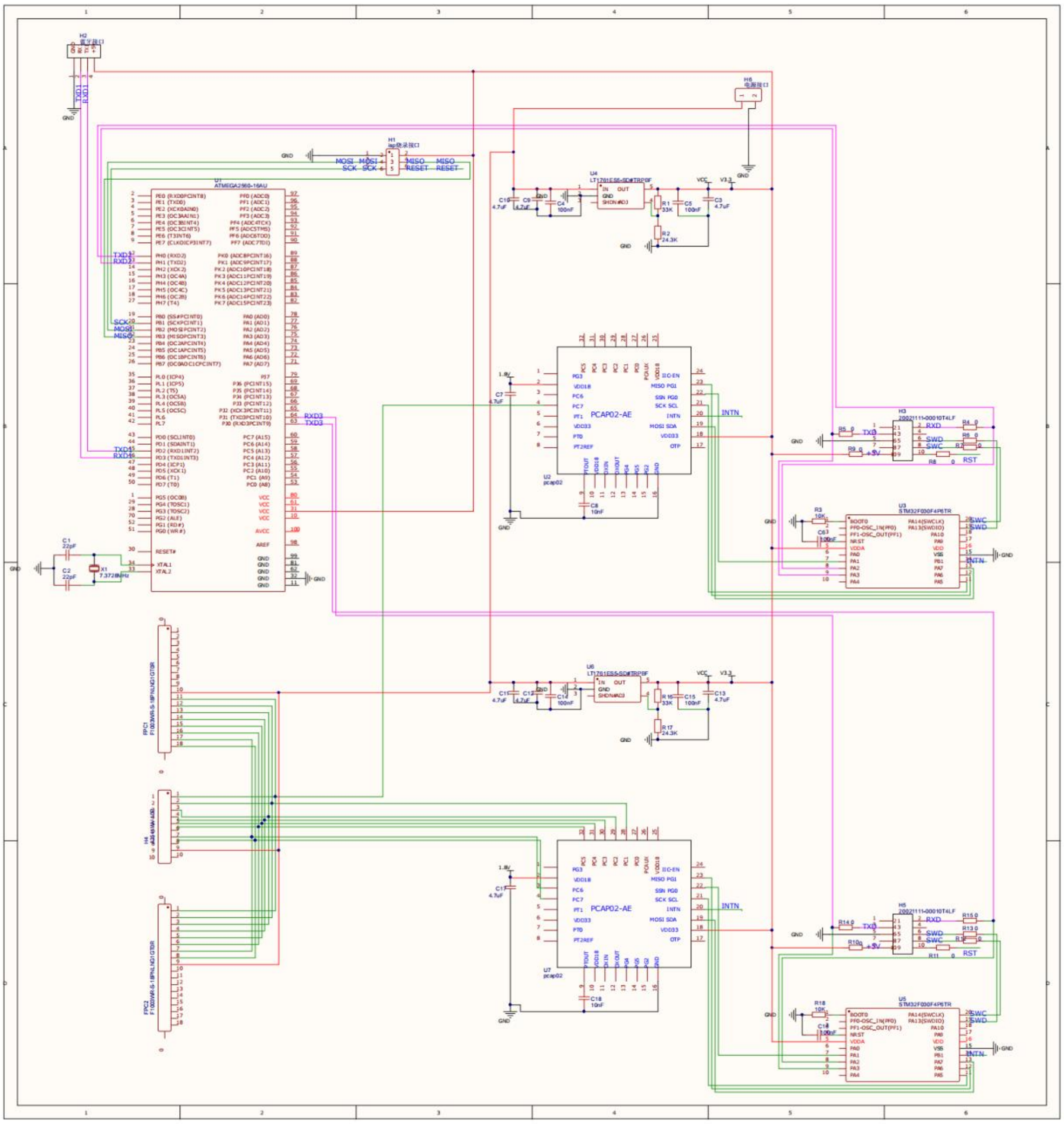

Figure S10. Detailed diagram of the circuit board.

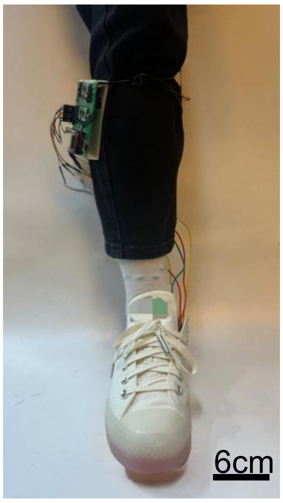

Figure S11. Optical photograph of the wearing demonstration.

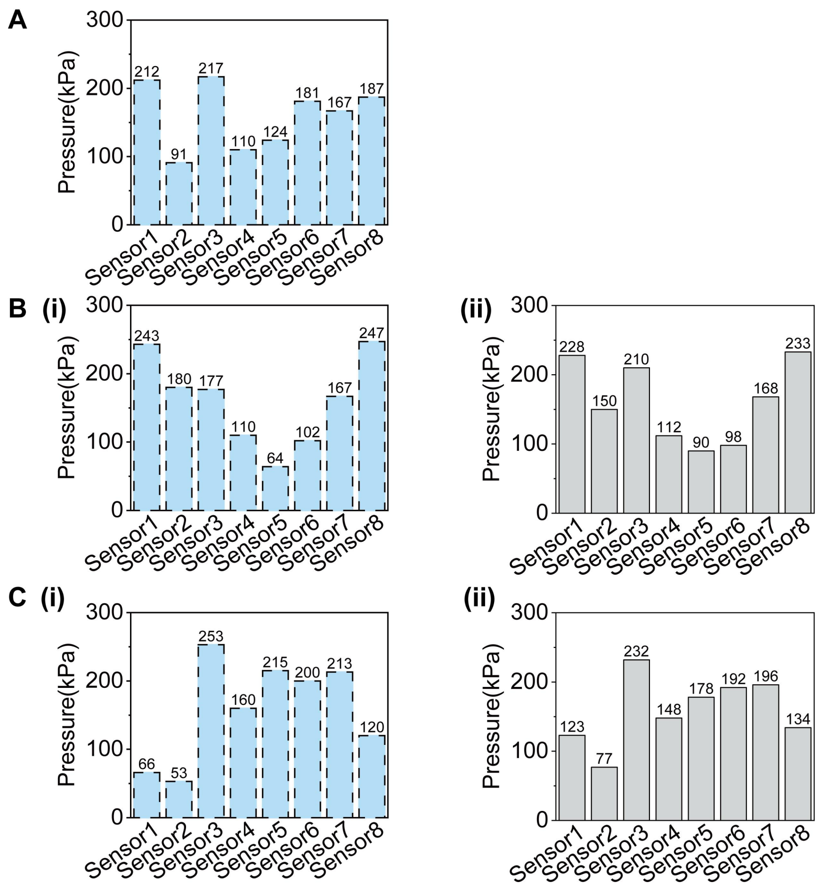

Figure S12. The corresponding pressure histogram of three standing postures. B) normal posture B) severe(i) and mild(ii) overpronation C) severe(i) and mild(ii) underpronation.

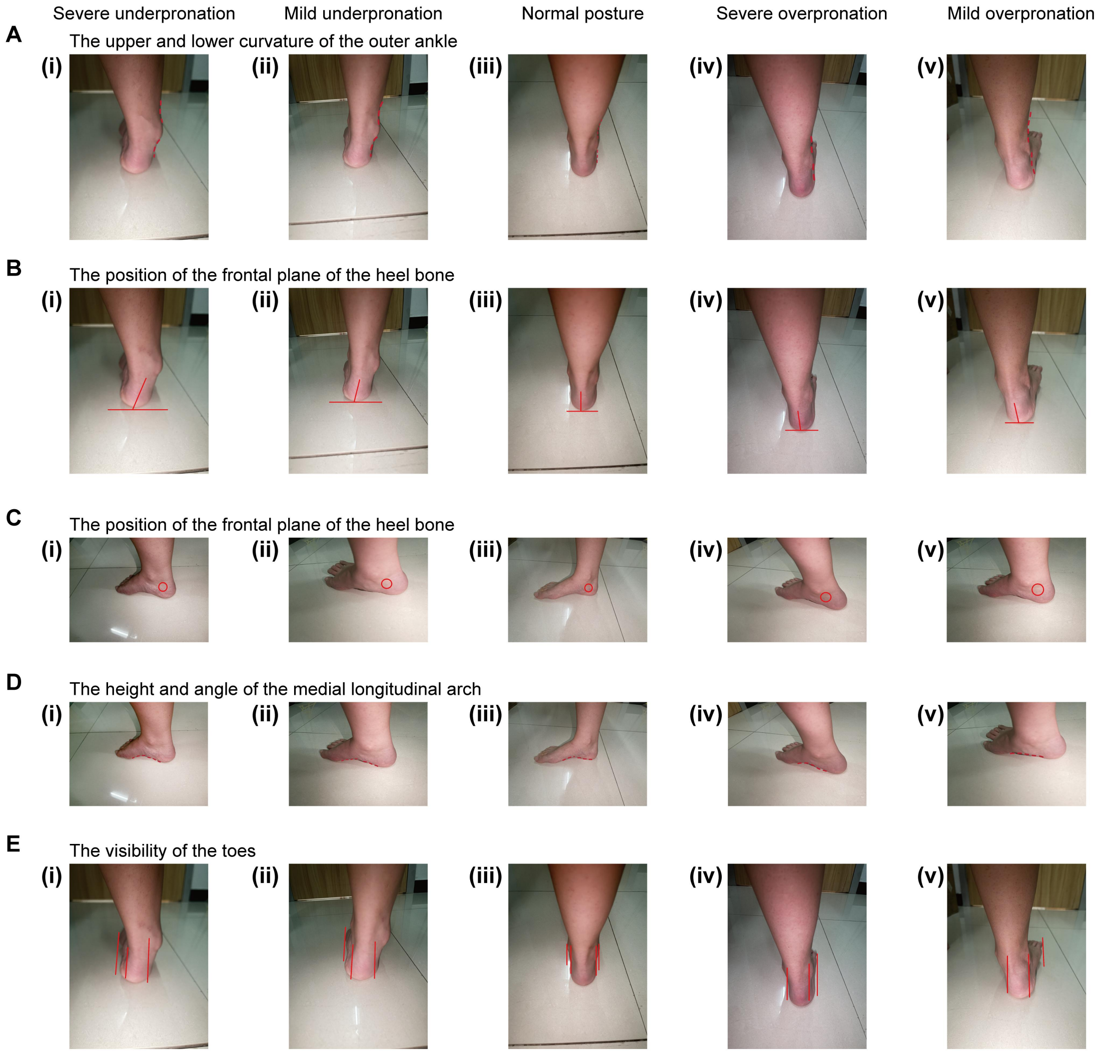

Figure S13. Evaluation and prediction of individual foot posture and foot and ankle function using the foot posture index[3](FPI) .The FPI is a criterion for evaluating and predicting individual foot posture and foot and ankle function in a clinical setting, including observation of the upper and lower curvature of the outer ankle (A) the position of the frontal plane of the heel bone (B) the elevation of the talonavicular region (C) the height and angle of the medial longitudinal arch (D) the visibility of the toes (E) distinguishing between five postures, including severe underpronatio(i) mild underpronatio(ii) normal posture(iii) severe overpronation(iv) and mild overpronation(v).

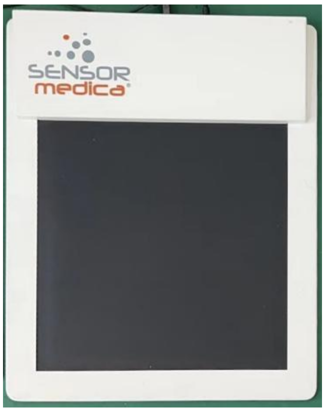

Figure S14. Optical photograph of the force plate (Sensor Medica).

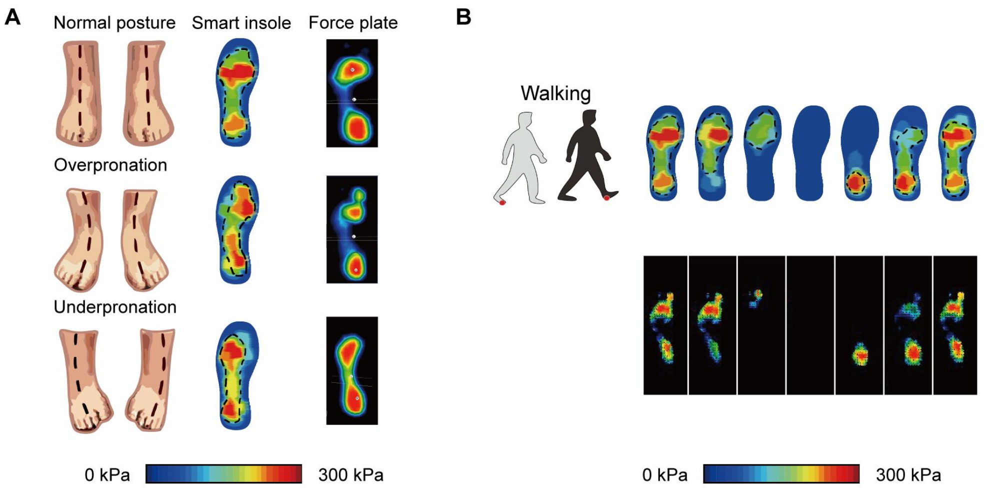

Figure S15. Comparison results of the smart insole and the force plate, including three static standing postures (A) and walking (B).

A

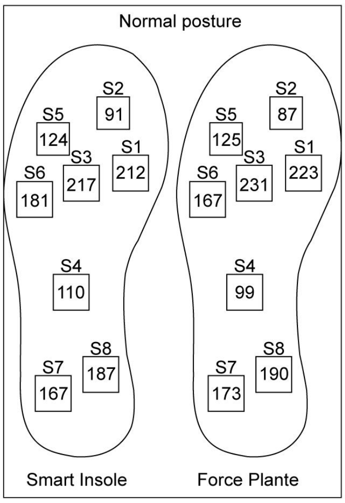

	Smart Insole	Standard Force Plate
Sensor 1	212	223
Sensor 2	91	87
Sensor 3	217	231
Sensor 4	110	99
Sensor 5	124	125
Sensor 6	181	167
Sensor 7	167	173
Sensor 8	187	190

B

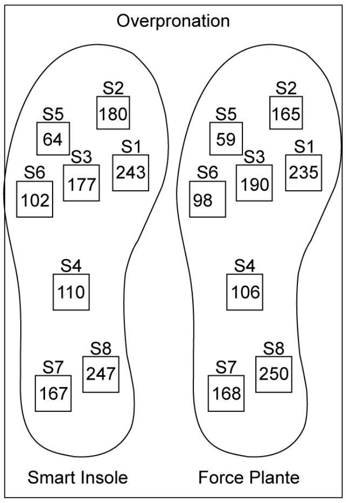

	Smart Insole	Standard Force Plate
Sensor 1	243	235
Sensor 2	180	165
Sensor 3	177	190
Sensor 4	110	106
Sensor 5	64	59
Sensor 6	102	98
Sensor 7	167	168
Sensor 8	247	250

C

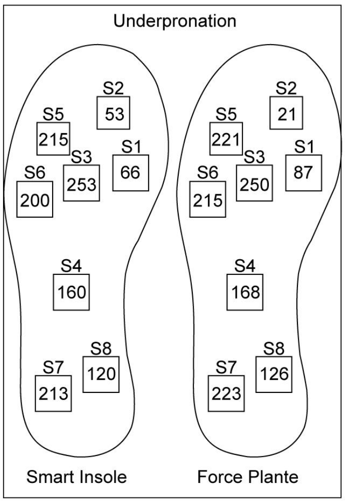

	Smart Insole	Standard Force Plate
Sensor 1	66	87
Sensor 2	53	21
Sensor 3	253	250
Sensor 4	160	168
Sensor 5	215	221
Sensor 6	200	215
Sensor 7	213	223
Sensor 8	120	126

Figure S16. Comparison of pressure values at 8 points between the smart insole and the standard force plate.

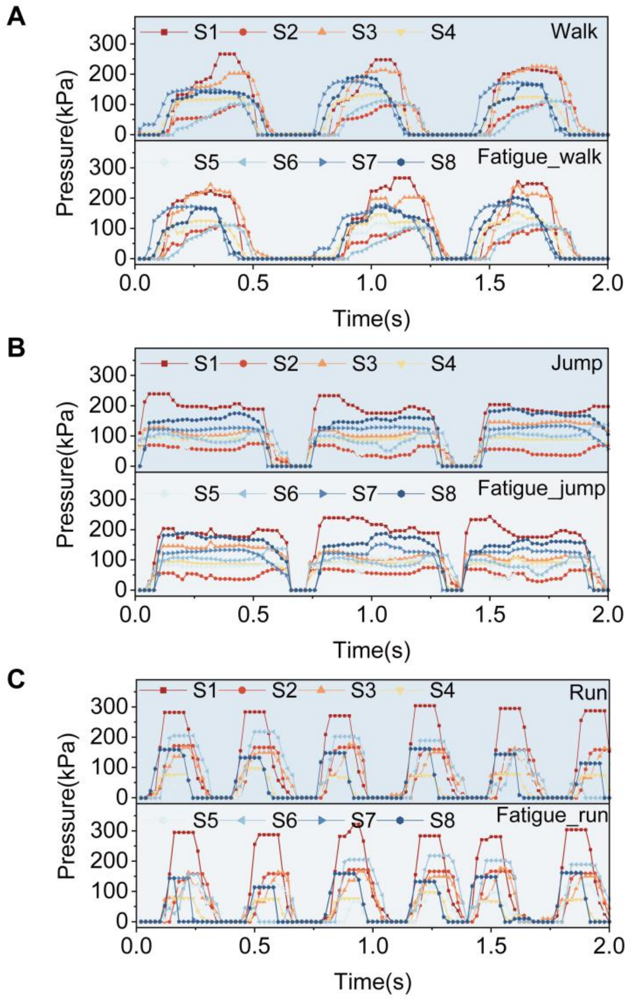

Figure S17. Capacitance response curves of the sensors as walking(A), jumping(B), and running(C) under the conditions of normal and fatigue by the experimenter.

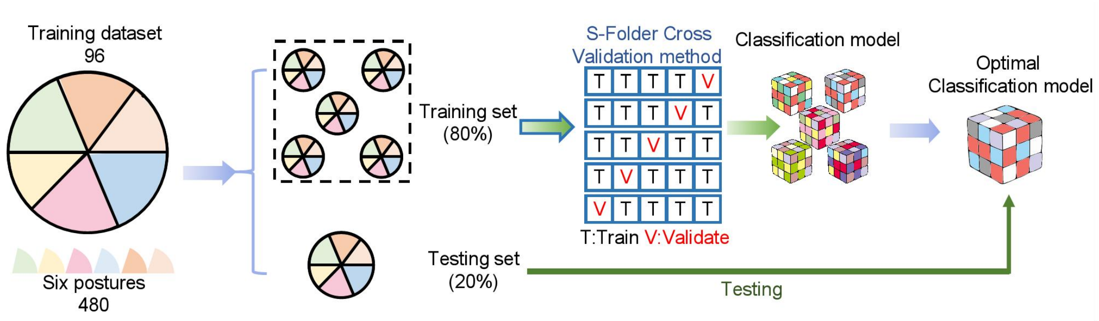

Figure S18. The training process of the 1D-CNN algorithm.

Table S1. Hicks, J. H. *et al.*[4] classified plantar pressure into 4 levels.

**Plantar pressure**	**Level**
0-50 kPa	Low pressure
50 kPa- 200 kPa	Medium pressure
200 kPa- 400 kPa	High pressure
>400 kPa	Very high pressure

Table S2. Basic information of the experimental participant.

**Experimental Participant Information**	
Gender	Female
Age	22
Height	162 cm
Weight	60 kg
Foot Size	24 cm
Athletic Ability	Yes
Health Condition	Good
Previous Foot Injuries	No

Table S3. Rate of Perceived Exertion (RPE) Scale.

RPE Scale	Rate of Perceived Exertion	Training Examples
1	Very Light Activity	Barely doing much of anything.
2-3	Light Activity	Very easy rides, walks, house hold chores.
4-6	Moderate Activity	Long steady aerobic building, body weight practice steady walking, hiking, comfortable running, etc.Working on moving efficiently and breathing
7-8	Vigorous Activity	Hiking moderate to difficult terrain, tempo riding strength & conditioning workout, trail run, challenging yard work.
9	Very Hard Activity	Max sustainable 60 minute effort, then you fall over. Can be 20 - 30 minutes max efforts held at a consistent pace. Difficult strength workout.
10	Max Effort Activity	Strength & Power Training. 30 seconds to 2 minute max efforts cycling or running.

References

[1] Z. Wei, Z. Zhang, J. Jiang, Y. Zhang, L. Wang, *Journal of Sports Sciences* **2019**, *37*, 2152-2158.

[2] R. Sanchis-Sanchis, C. Blasco-Lafarga, A. Encarnacion-Martinez, P. Perez-Soriano, *Gait & Posture* **2020**, *77*, 250-256.

[3] R. Scharfbillig, A. M. Evans, A. W. Copper, M. Williams, S. Scutter, H. Iasiello, A. Redmond, *Journal of the American Podiatric Medical Association* **2004**, *94*, 31-38.

[4] J. H. Hicks, *Journal of anatomy* **1954**, *88*, 25-30.
